# The Impact of Single Session Intermittent Theta-Burst Stimulation over the Dorsolateral Prefrontal Cortex and Posterior Superior Temporal Sulcus on Adults with Autism Spectrum Disorder

**DOI:** 10.3389/fnins.2017.00255

**Published:** 2017-05-09

**Authors:** Hsing-Chang Ni, June Hung, Chen-Te Wu, Yu-Yu Wu, Chee-Jen Chang, Rou-Shayn Chen, Ying-Zu Huang

**Affiliations:** ^1^Department of Psychiatry, Chang Gung Memorial Hospital at LinkouLinkou, Taiwan; ^2^Graduate Institute of Clinical Medicine, National Taiwan University College of MedicineTaipei, Taiwan; ^3^Department of Neurology, Neuroscience Research Center, Chang Gung Memorial Hospital at LinkouTaipei, Taiwan; ^4^Department of Medical Imaging and Intervention, Chang Gung Memorial Hospital at LinkouTaipei, Taiwan; ^5^Graduate Institute of Clinical Medical Science, Chang Gung UniversityTaoyuan, Taiwan; ^6^Clinical Informatics and Medical Statistics Research Center, Chang Gung UniversityTaoyuan, Taiwan; ^7^Research Services Center for Health Information, Chang Gung UniversityTaoyuan, Taiwan; ^8^Medical School, Chang Gung UniversityTaoyuan, Taiwan; ^9^Institute of Cognitive Neuroscience, National Central UniversityTaoyuan, Taiwan

**Keywords:** repetitive transcranial magnetic stimulation, theta burst stimulation, dorsolateral prefrontal cortex, posterior superior temporal sulcus, autism spectrum disorder

## Abstract

Intermittent theta burst stimulation (iTBS), a patterned repetitive transcranial magnetic stimulation, was applied over the posterior superior temporal sulcus (pSTS) or dorsolateral prefrontal cortex (DLPFC) to explore its impact in adults with autism spectrum disorder (ASD). Among 25 adults with ASD, 19 (mean age: 20.8 years) completed the randomized, sham-controlled, crossover trial. Every participant received iTBS over the bilateral DLPFC, bilateral pSTS and inion (as a sham control stimulation) in a randomized order with a 1-week interval. Neuropsychological functions were assessed using the Conners' Continuous Performance Test (CCPT) and the Wisconsin Card Sorting Test (WCST). Behavioral outcomes were measured using the Yale-Brown Obsessive Compulsive Scale (Y-BOCS) and the Social Responsiveness Scale (SRS). In comparison to that in the sham stimulation, the reaction time in the CCPT significantly decreased following single DLPFC session (*p* = 0.04, effect size = 0.71) while there were no significant differences in the CCPT and WCST following single pSTS session. Besides, the results in behavioral outcomes were inconsistent and had discrepancy between reports of parents and patients. In conclusion, a single session of iTBS over the bilateral DLPFC may alter the neuropsychological function in adults with ASD. The impacts of multiple-sessions iTBS over the DLPFC or pSTS deserve further investigations.

## Introduction

Autism spectrum disorder (ASD) is characterized by an early onset of difficulties with social-communication, and repetitive, restricted, stereotyped behaviors, and interests (Lai et al., 2014). Although the precise etiology of ASD is not conclusive, abnormalities in regional brain volumes (Nickl-Jockschat et al., 2012), patterns of brain perfusion (Ohnishi et al., 2000), neural biochemical characteristics of ASD (Baruth et al., 2013), and excitatory/inhibitory neurotransmission (LeBlanc and Fagiolini, 2011) have been reported. Unfortunately, there are limited biological interventions available to treat ASD.

Repetitive transcranial magnetic stimulation (rTMS), a non-invasive technique of repetitive stimulation of the neural circuits, is capable of producing long-lasting changes in cortical excitability beyond the period of stimulation (Fang et al., 2010; Pell et al., 2011). Although the precise mechanism of action of rTMS is still unclear, recent studies found that rTMS can induce changes similar to long-term potentiation (LTP) or long-term depression (LTD) via modulation of synaptic plasticity (Huang et al., 2007; Pell et al., 2011). Recently, rTMS has shown some potential clinical benefits in several psychiatric disorders such as major depressive disorder (Gaynes et al., 2014), schizophrenia (Rajji et al., 2013), post-traumatic stress disorder (Cohen et al., 2004; Watts et al., 2012), and ASD (Enticott et al., 2012, 2014).

The application of rTMS in ASD has been studied recently. Several neurobiological targets, including the dorsolateral prefrontal cortex (DLPFC), medial prefrontal cortex, dorso-medial prefrontal cortex and Broca's area, have been studied (Sokhadze et al., 2009, 2010, 2012; Baruth et al., 2010; Enticott et al., 2011, 2012, 2014; Fecteau et al., 2011). Among these, rTMS over the DLPFC has been investigated the most. Studies have investigated the therapeutic effect of rTMS over the DLPFC using several different designs, such as changes in the stimulation site (left, right, or bilateral), stimulation frequency (from once per week to twice per week), total number of stimulation sessions (1–18) and protocol frequency (0.5–5 Hz) (Oberman et al., 2016). These studies demonstrated that low-frequency rTMS over the DLPFC might restore the excitatory/inhibitory imbalance and improve the repetitive behaviors observed in ASD. However, the social impairments observed in ASD did not change with these interventions (Baruth et al., 2010; Sokhadze et al., 2010, 2014; Casanova et al., 2014).

Another potential target of rTMS for ASD is the posterior superior temporal sulcus (pSTS). The pSTS was first found to be involved in the process of biological motion (Puce and Perrett, 2003) and a further important role for it has been demonstrated in social perception (Zilbovicius et al., 2006; Redcay, 2008). Atypical activation patterns of the pSTS in ASD have been reported in several social experiments (Mason et al., 2008; Redcay et al., 2013). Moreover, studies on cerebral blood flow revealed hypoperfusion of the bilateral STS in sedated children with ASD (Ohnishi et al., 2000; Zilbovicius et al., 2000) and the level of hypoperfusion in the left STS was associated with the clinical severity of autism (Gendry Meresse et al., 2005).

The impact of rTMS over the pSTS has been tested in healthy adults. Grossman first found that low-frequency rTMS over the right pSTS temporarily impaired the perception of biological motion in healthy adults (Grossman et al., 2005). In addition, van Kemenade demonstrated that the sensitivity to detect biological motion marginally declined after continuous theta burst TMS over the left pSTS (van Kemenade et al., 2012). One recent fMRI study demonstrated that low-frequency rTMS over the bilateral pSTS produced remote hemodynamic effects in a network of specific brain areas, including the lateral occipital-temporal cortex, intraparietal sulcus, and ventral premotor cortex (Arfeller et al., 2013). Since these studies demonstrated that low-frequency rTMS (inhibitory protocol) over the pSTS might impair the perception of biological motion in healthy adults, it might be possible to apply an excitatory protocol over the pSTS to enhance the perception of biological motion. Therefore, it might be feasible to enhance the function of the pSTS to improve social cognition in patients with ASD (Saitovitch et al., 2016). However, to the best of our knowledge, there has so far been no study investigating the impact of rTMS over the pSTS in patients with ASD.

Theta burst stimulation (TBS), a modified protocol of rTMS, can very quickly produce an LTP- or LTD-like effect by using bursts at the same frequency (three pulses at 50 Hz, repeated five times per second) and intensity (Huang et al., 2005, 2007, 2011). For TBS, the direction of the after-effects depends on whether the bursts are delivered continuously (cTBS, producing LTD-like effects, inhibitory) or intermittently (iTBS, producing LTP-like effects, excitatory). In comparison to traditional rTMS, the stimulus duration is shorter in TBS (TBS: 20–240 s; rTMS: 5–30 min) (Pell et al., 2011). The shorter stimulus duration makes TBS more appropriate for ASD in clinical practice.

In this pilot study, we aimed to explore the impacts of single session TBS over the DLPFC and the pSTS in ASD. Based on the review mentioned above, the excitatory protocol (iTBS) was chosen for use in our study. In addition, bilateral cerebral hemispheres were stimulated to maximize the intervention effect. Using a randomized, crossover, and sham-controlled study design, we investigated the impact of iTBS over the bilateral DLPFC and bilateral pSTS compared to the sham-control condition. Neuropsychological function was measured using the Conner's Continuous Performance Test (CCPT) and the Wisconsin Card Sorting Test (WCST). In addition, the behavioral outcomes were measured using the Yale-Brown Obsessive Compulsive Scale (Y-BOCS) and the social responsiveness scale (SRS) in both the participants and their parents.

## Materials and methods

### Subjects

We recruited participants, who were older than 18 years of age through advertisement at the outpatient clinic of the Department of Psychiatry, Chang Gung Memorial Hospital, Taiwan. People interested in our study were first interviewed by a board-certified child psychiatrist (First author). Once they and their parents agreed to join the study, several assessments were arranged. First, the diagnosis of ASD was evaluated according to the DSM-IV and ICD-10 criteria by the first author and this were further confirmed using the Chinese version of the Autism Diagnostic Interview-Revised (ADI-R) and Autism Diagnostic Observation Schedule (ADOS) by another senior board-certified child psychiatrist (Dr. Wu). Patients with a diagnosis of autistic disorder, Asperger syndrome, or pervasive developmental disorder, were included in our study. Patients with any history of systemic medical illness, seizures, severe head injury, suicide attempts, schizophrenia, bipolar affective disorder, substance abuse, pregnancy, or the presence of an implanted medical device such as a cardiac pacemaker, were excluded from the study. A total of 25 participants with ASD were enrolled in the current study. Six participants completed the baseline assessments but refused the iTBS interventions for personal reasons. The other 19 participants, aged 18–29 years old, completed the entire study. The demographic data are shown in Table 1. The participants were maintained on the same medication during the whole study period. Three of the participants (16%) took psychotropic medications (methylphenidate = 1, fluoxetine = 1, and sertraline = 1).

**Table 1:** **Demographic data of participants**.

	**ASD (*n* = 19)**
Age, mean (SD)	20.8 (1.4)
Sex, male (%)	14 (73.7)
Education level (n)	
Senior high school or lower	7
College	10
Graduate school or higher	2
Full-scale IQ, Mean (SD)	100.5 (14.0)
Verbal IQ, mean (SD**)**	96.9 (16.5)
Performance IQ, mean (SD)	103.8 (13.4)
ADOS	
Language and communication, mean (SD)	4.0 (1.3)
Reciprocal social interaction, mean (SD)	6.7 (1.9)
Imagination, mean (SD)	1.1 (0.5)
Stereotyped behaviors and restricted interests, mean (SD)	1.4 (0.7)
ADI-R	
Social, mean (SD)	19.3 (5.4)
Communication, mean (SD)	21.6 (7.1)
Repetitive and stereotyped behavior, mean (SD)	6.5 (2.3)
Psychotropic Medications (n)	
Sertraline (20 mg/day)	1
Fluoxetine (40 mg/day)	1
Methylphenidate (20 mg/day)	1

*ASD, autism spectrum disorder; ADOS, Autism Diagnostic Observation Schedule; ADI-R, Autism Diagnostic Interview-Revised*.

### Study design

This was a randomized, sham-controlled, crossover trial to investigate the impact of iTBS in ASD. The participants received iTBS over the bilateral DLPFC, bilateral pSTS and inion (as a sham control stimulation) in a randomized order, with a 1-week interval between each session. The order of randomization of the DLPFC, pSTS, and inion followed the rules of William design with an order of 3 (6 sequences). The social and repetitive behaviors were assessed immediately before, 8 h and 2 days after the interventions. Neuropsychological functions were assessed before and after (within 1 h) the intervention.

The Research Ethics Committee at the Chang Gung Memorial Hospital approved this study before its implementation. The procedures and purpose of our study were explained face-to-face to the participants and their parents, who then provided written informed consent.

### Target identification

The scans were collected on a 3T magnetic resonance imaging (MRI) scanner (Trio, Siemens Medical Solutions, Erlangen, Germany), using a 12-channel head coil. A high resolution 3D-MPRAGE sequence for anatomic localization was acquired using the following parameters: TR = 2,250 ms, TE = 2.6 ms, TI = 900 ms, FOV = 240 mm, Flip Angle = 9°, matrix = 240 × 256, voxel size = 1.0 × 1.0 × 1.0 mm. To minimize movement artifacts, the head of every subject was firmly fixed with pads. All images were examined to ensure that they were free of motion and artifacts at the time of image acquisition.

We derived the iTBS sites for each of our individual participants using the same normalized coordinates. The sites of the left and right pSTS were based on the study by Van Overwalle and Baetens (2009), who analyzed 200 functional MRI (fMRI) studies and reported the averaged pSTS coordinates on the Talairach atlas (±50, −55, 10). The final Montreal Neurological Institute (MNI) coordinates of the pSTS were ±50.5, −57.1, 7.9 (for conversion from the coordinates on the Talairach atlas to those on the MNI template, refer to http://imaging.mrc-cbu.cam.ac.uk/imaging/MniTalairach). The sites of the left and right DLPFC (MNI coordinates: −41.9, 35.1, 33.7; 39.9, 36.8, 33.9, respectively) were adapted from Mylius et al. (2013), where researchers first anatomically defined the DLPFC at the separating line between the anterior and middle thirds of the middle frontal gyrus, and then derived the MNI coordinates through structural normalization using 50 normal volunteers (24 men). We specifically used a subset of data from male participants here since our present experiment happened to recruit mainly male participants.

**Figure 1:**
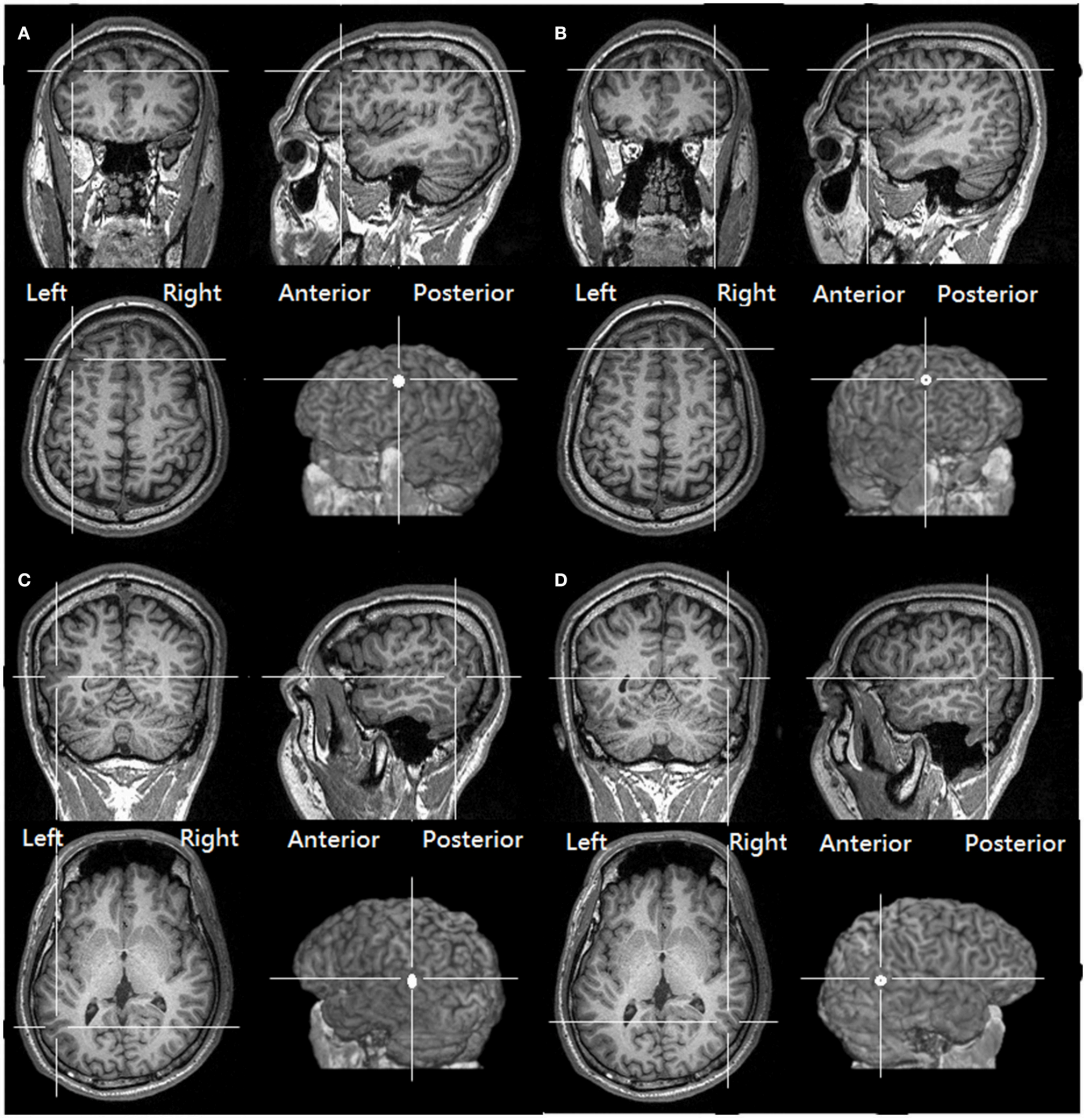
**The targeted location over the (A)** left DLPFC **(B)** right DLPFC **(C)** Left pSTS **(D)** right pSTS of one of our participants, as derived from transforming the same normalized MNI coordinates to the structural image of the participant (See Methods, Target identification).

To target the normalized coordinates precisely, the T1-weighted structural image of each participant was first spatially normalized with SPM8 (http://www.fil.ion.ucl.ac.uk/spm/software/spm8) to find the transformation matrix. We then applied an inverse transformation matrix, which accepts the normalized coordinates as the input and gives the corresponding coordinates in the participant's native structural image as the output (Figure 1 shows the four stimulation sites of a representative participant and the coordinates rendered on a normalized skull-stripped brain). These output coordinates were marked on the native structural image in the Navigated Brain Stimulation (NBS) system (Nexstim®, Helsinki, Finland), which, with the aid of an infrared tracking device, can visualize the position of the TMS coil relative to the structure of the head and brain of each individual.

### Transcranial magnetic stimulation

Electromyography was recorded with surface electrodes from the right first dorsal interosseous muscle (FDI) using a belly-tendon montage. TMS was performed using a 70 mm figure-of-eight coil connected to a Magstim Super Rapid^2^ system (Magstim Company, Oxford, UK). The coil was placed over the contralateral motor cortex tangentially to the scalp with the handle pointing backward. The motor hot-spot was determined as the location on the scalp where TMS produced the largest MEP from the FDI at rest. The active motor threshold (AMT) was defined as the minimum stimulation intensity over the motor hot-spot that could elicit an MEP of no less than 200 mV in 5 out of 10 trials during voluntary contraction of the FDI.

### Theta burst stimulation

The iTBS paradigm, which has been shown to produce long-lasting excitation of the cortex, was applied in the current study (Huang et al., 2005). Each TBS train comprised of a burst of 3 pulses at 50 Hz given 10 times every 200 ms. The TBS train was given 20 times every 10 s so that there were 600 pulses in total. In the study, two courses of iTBS separated by a 5-min break were given to the target in the left hemisphere first. Then, two other courses of iTBS were applied to the target in the right hemisphere. With regard to the inion, two courses of iTBS were given with a break of 5 min.

The stimulus intensity for the DLPFC and pSTS was set at 80% AMT of the right FDI. As for the stimulus intensity for the sham control stimulation over the inion, 60% of the AMT was applied with an 180-degree reversal of the coil (Huang et al., 2012; Chuang et al., 2014).

### Neuropsychological function outcome measures

The CCPT and WCST, which were used to evaluate the impact of iTBS on neuropsychological function, were completed before and within 1 h after the stimulation.

The CCPT is a non-X type CPT test, which is a Go/No-Go task lasting for 14 min (Conners et al., 2003). For the test, 360 trials, which took approximately 250 ms each, appeared on the computer screen. The participant was requested to respond by pressing the spacebar on the keyboard when a letter other than the target letter “X” appeared. The reaction times, omission errors, and commission errors were calculated to assess neuropsychological function before and after TBS.

The computerized version of the WCST: Computer Version 4 research edition (WCST: CV4) (Heaton, 2003) was used in our study. The participants were asked to choose the correct card from four categories of cards, in which the stimuli are multidimensional according to color, shape, and number and each dimension is defined by a sorting rule. By trial and error, the participant had to find the sorting rule given from the feedback (“Right” or “Wrong”) on the screen following each sort. The classification principle changed without warning following sequences of 10 consecutive correct matches. Testing continued until all 128 cards were sorted and irrespective of whether the participant completed all of the rule shifts. We measured the total errors, perseverative response (the number of incorrect responses that had been correct for the preceding category), perseveration errors (the number of errors where the participant used the same rule for their choice as the previous choice), conceptual level responses (the number of correct responses that occurred in runs of three or more divided by the number of trials and then multiplied by 100), and the number of categories completed (the number of runs of ten corrected responses).

### Clinical symptoms outcome measures

The Y-BOCS and SRS were used to evaluate the impact of iTBS on behavioral outcomes. The Y-BOCS and SRS were completed by participants and their parents three times, i.e., before, 8 h and 2 days after the iTBS conditioning, for every stimulation session.

The SRS is a self-report instrument that quantifies the severity of social communication deficits in ASD (Constantino, 2005). The SRS contains 65 items and can be completed by the participants or their parents in approximately 15 min. Items were rated on a 4-point Likert scale from “1” (not sure) to “4” (almost always true) and can be rated in five subscales: social awareness, social cognition, social communication, social motivation, and autistic mannerisms. Previous studies have demonstrated the good psychometric properties of the SRS in children, adolescents, and adults (Constantino, 2005; Constantino and Todd, 2005; Bolte, 2012). In addition, the psychometric properties of the Chinese version of the SRS were also investigated and they demonstrated good test–retest reliability (intra-class correlations: 0.751–0.852), internal consistency (Cronbach's alpha: 0.944–0.947), and convergent validity with the Chinese SCQ (Pearson correlations: 0.609–0.865) (Gau et al., 2013).

The Y-BOCS is a gold-standard measurement tool for symptoms severity in obsessive-compulsive disorder (Goodman et al., 1989a,b), and can be used to assess the repetitive behaviors observed in ASD (Hollander et al., 2000, 2006). The Y-BOCS can be assessed by clinicians or by self-report (Baer, 1991). The Y-BOCS consists of a comprehensive symptom checklist and a 10-item severity scale. The severity scale evaluates the recent degree of impairments in five clinical domains: time taken, functional impairment, psychological distress, efforts to resist, and perceived sense of control. Items are rated on a 5-point Likert scale (range: 0–4) and are used to generate a total Y-BOCS score and subscale scores for obsessions and compulsions. Previous studies have demonstrated the good psychometric properties of the Y-BOCS (Frost et al., 1995; Woody et al., 1995).

### Statistical analysis

The means (±standard deviation, SD) of the behavioral and neuropsychological outcomes were calculated for each iTBS intervention. The changes of outcomes were compared using a linear mixed model for repeated measures to analyze the group difference (DLPFC vs. sham, pSTS vs. sham) within one model. In the linear mixed model, we used the study periods and intervention group as fixed effects and the participants as a random effect. The t-type confidence limits were constructed for each of the fixed-effects parameter estimates. *P*-values were adjusted using Dunnett's test for the comparison of intervention groups and sham control. In addition, Cohen's d was used to compute the effect size on the changes from baseline for the comparison of intervention groups and sham control. A *p* < 0.05 was considered significant. SAS 9.3 software was used for our analysis.

## Results

### Neuropsychological functions

The raw data of the neuropsychological outcomes before and after iTBS are presented in Table 2. In summary, omission errors and reaction time decreased after iTBS over the DLPFC, while commission errors increased after iTBS over the pSTS in the CCPT. As for the WCST, total errors, perseveration responses and errors decreased after the sham stimulation, but there was no obvious difference in the DLPFC and pSTS interventions.

**Table 2:** **Neuropsychological outcome before and after interventions**.

	**DLPFC**			**pSTS**			**Sham**		
	**Baseline mean (SD)**	**Post 1 h mean (SD)**	**Difference mean (SD)**	**Baseline mean (SD)**	**Post 1 h mean (SD)**	**Difference mean (SD)**	**Baseline mean (SD)**	**Post 1 h mean (SD)**	**Difference mean (SD)**
**CCPT**									
Reaction time (ms)	404.99 (101.69)	372.11 (57.90)	−32.89 (81.99)	398.67 (85.49)	389.82 (87.29)	−8.85 (35.03)	381.17 (62.41)	382.75 (67.77)	1.58 (21.91)
Omission error	5.26 (12.61)	2.68 (6.63)	−2.58 (8.51)	3.79 (7.48)	3.16 (6.36)	−0.63 (3.61)	3.37 (6.39)	4.26 (9.78)	0.89 (7.21)
Commission error	15.47 (10.05)	14.68 (9.27)	−0.79 (5.76)	14.16 (9.31)	16.63 (8.92)	2.47 (3.37)	16.79 (9.75)	15.79 (9.79)	−1.00 (5.46)
**WCST**									
Total errors	12.53 (6.74)	11.21 (8.24)	−1.32 (7.51)	11.47 (6.60)	11.58 (8.66)	0.11 (5.23)	17.11 (14.48)	11.21 (7.49)	−5.89 (13.29)
Perseveration response	6.47 (4.51)	6.00 (3.71)	−0.47 (3.79)	6.47 (5.90)	6.68 (5.87)	0.21 (5.13)	8.74 (7.61)	5.84 (4.11)	−2.89 (7.13)
Perseveration errors	6.11 (3.54)	5.68 (3.20)	−0.42 (3.04)	6.11 (4.78)	6.26 (4.62)	0.16 (3.95)	8.05 (6.32)	5.58 (3.53)	−2.47 (5.75)
Conceptual level response	68.21 (6.69)	70.37 (6.71)	2.16 (10.09)	69.74 (7.33)	67.47 (6.92)	−2.26 (6.36)	71.00 (15.34)	68.53 (6.18)	−2.47 (15.37)
Number of category completed	6.00 (0.00)	5.95 (0.23)	−0.05 (0.23)	5.89 (0.46)	5.74 (0.81)	−0.16 (0.69)	5.95 (0.23)	6.00 (0.00)	0.05 (0.23)

*CCPT, Conner's Continuous Performance Test; WCST, Wisconsin Card Sorting Test*.

When comparing the pSTS and DLPFC iTBS treatments to sham treatments (Table 3), the reaction time in the CCPT significantly decreased after iTBS over the DLPFC (*p* = 0.04, effect size = 0.71), while commission errors in the CCPT, and total errors in the WCST non-significantly increased after iTBS over the pSTS (*p* = 0.07, effect size = 0.79; *p* = 0.06, effect size = 0.65, respectively).

**Table 3:** **Adjusted estimate of neuropsychological outcome difference from baseline based on the mixed model analysis**.

	**Post1h_basline**									
	**DLPFC vs. Sham**					**pSTS vs. Sham**				
**Outcome**	**Estimate (95% CI)**	**Degrees of freedom**	**t statistic**	***p*-value**	**Cohen's d**	**Estimate (95% CI)**	**Degrees of freedom**	**t statistic**	***p*-value**	**Cohen's d**
**CCPT**										
Omissions	−3.60 (−8.10 to 0.89)	34	−1.57	0.22	0.44	−1.59 (−6.09 to 2.91)	34	−0.69	0.71	0.28
Commissions	0.22 (−2.86 to 3.29)	34	0.14	0.99	0.04	3.38 (0.30 to 6.45)	34	2.15	0.07	0.79
Hit RT	−38.16 (−69.58 to −6.74)	34	−2.38	0.04^*^	0.71	−13.26 (−44.74 to 18.21)	34	−0.83	0.62	0.55
**WCST**										
Total errors	4.17 (−0.65 to 8.99)	34	1.69	0.17	0.44	5.55 (0.73 to 10.37)	34	2.25	0.06	0.65
Perseverative responses	2.15 (−0.94 to 5.24)	34	1.36	0.30	0.44	2.84 (−0.25 to 5.94)	34	1.8	0.14	0.51
Perseverative errors	1.84 (−0.60 to 4.28)	34	1.48	0.25	0.47	2.41 (−0.03 to 4.85)	34	1.94	0.11	0.54
Conceptual level responses	4.68 (−2.86 to 12.21)	34	1.22	0.38	0.36	0.30 (−7.24 to 7.84)	34	0.08	0.99	0.02
Categories completed	−0.29 (−0.68 to 0.09)	34	−1.49	0.17	0.34	−0.41 (−0.79 to −0.02)	34	−2.06	0.15	0.37

The mixed model analysis was adjusted by sequence and period.

* *p < 0.05*.

*CCPT, Conner's Continuous Performance Test; WCST, Wisconsin Card Sorting Test*.

### Clinical symptoms

**Figure 2:**
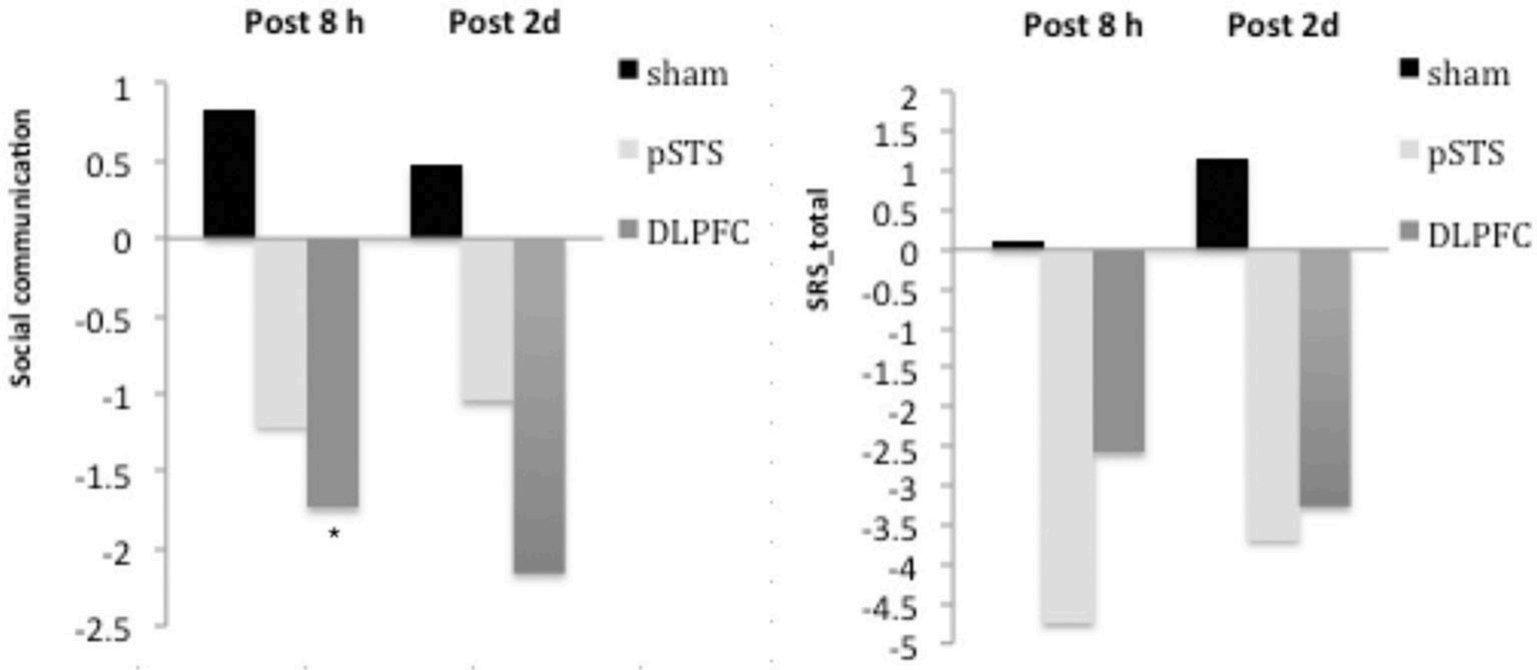
**Changes of behavioral symptoms before and after iTBS using participants' reports**. ^*^p < 0.05.

**Figure 3:**
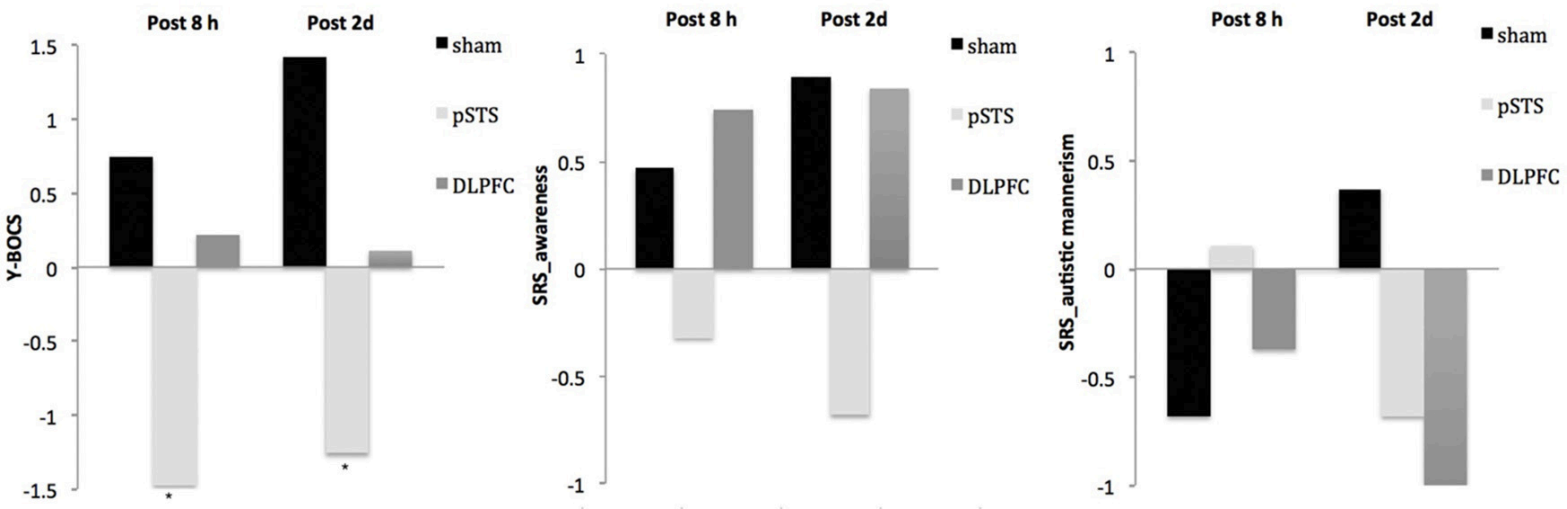
**Changes of behavioral symptoms before and after iTBS using parents' reports**. ^*^p < 0.05.

The raw data of clinical outcomes including baseline, 8 h post-iTBS (post 8 h) and 2 days post-iTBS (post 2 days) are presented in Table 4. The outcome differences (before and after interventions) in the DLPFC and pSTS in comparison to the sham treatment are presented in Table 5 (post 8 h) and Table 6 (post 2 days). The differences were presented in Figure 2 (participants) and Figure 3 (parents).

**Table 4:** **Behavioral outcomes at baseline, post 8 h and post 2 days reported by participants and parents**.

		**DLPFC**			**pSTS**				**Sham**	
**Outcomes**		**Baseline**	**Difference (Post8h-Baseline)**	**Difference (Post2d-Baseline)**	**Baseline**	**Difference (Post8h-Baseline)**	**Difference (Post2d-Baseline)**	**Baseline**	**Difference (Post8h-Baseline)**	**Difference (Post2d-Baseline)**
		**Mean (SD)**	**Mean (SD)**	**Mean (SD)**	**Mean (SD)**	**Mean (SD)**	**Mean (SD)**	**Mean (SD)**	**Mean (SD)**	**Mean (SD)**
Participant	Y-BOC: Obsession	5.00 (3.89)	−1.63 (2.27)	−1.32 (1.86)	4.32 (4.41)	−0.47 (1.07)	−0.47 (1.61)	4.58 (4.67)	−1.00 (2.79)	−1.26 (2.62)
	Y-BOC: Compulsion	2.79 (3.24)	−0.26 (1.56)	−0.11 (1.41)	2.58 (3.37)	0.16 (1.83)	0.11 (1.56)	2.68 (3.23)	0.32 (2.94)	0.26 (2.94)
	SRS total	87.26 (32.49)	−2.58 (10.90)	−3.26 (11.94)	89.0 (26.22)	−4.74 (7.37)	−3.68 (8.86)	84.63 (28.12)	0.11 (6.35)	1.16 (8.07)
	Awareness	9.79 (4.60)	−0.16 (2.41)	−0.58 (2.17)	9.89 (3.93)	−0.26 (2.60)	0.00 (2.00)	9.53 (4.02)	−0.47 (2.22)	−0.58 (2.55)
	Cognition	16.32 (5.31)	−0.16 (2.77)	0.05 (3.12)	17.32 (4.26)	−0.84 (2.12)	−1.53 (3.41)	16.84 (4.84)	−0.58 (2.39)	−0.11 (2.77)
	Communication	29.58 (11.62)	−1.74 (3.91)	−2.16 (4.39)	29.84 (10.38)	−1.21 (3.65)	−1.05 (3.61)	27.63 (10.90)	0.84 (2.87)	0.47 (3.96)
	Motivation	17.00 (7.21)	0.21 (2.99)	−0.16 (3.75)	17.11 (6.18)	−0.63 (2.91)	0.11 (2.77)	16.21 (6.09)	0.68 (2.54)	1.26 (2.23)
	Autistic mannerisms	14.58 (8.02)	−0.74 (3.68)	−0.42 (2.55)	14.89 (7.22)	−1.79 (3.07)	−1.21 (2.64)	14.42 (6.79)	−0.37 (2.29)	0.11 (2.79)
Parents	Y-BOCS: Compulsion	3.89 (3.95)	0.22 (1.31)	0.11 (1.08)	5.16 (4.49)	−1.47 (3.37)	−1.26 (3.51)	2.68 (3.13)	0.74 (1.59)	1.42 (2.80)
	SRS total	97.63 (29.65)	0.68 (11.03)	−0.68 (11.70)	95.47 (30.34)	0.16 (12.17)	−0.05 (9.63)	94.32 (28.08)	0.16 (9.97)	3.74 (11.58)
	Awareness	11.00 (3.25)	0.74 (2.05)	0.84 (2.14)	11.58 (3.19)	−0.32 (1.80)	−0.68 (1.53)	10.89 (3.43)	0.47 (2.32)	0.89 (2.58)
	Cognition	20.68 (5.10)	−0.79 (2.80)	−0.79 (3.21)	18.95 (5.63)	0.16 (2.29)	0.58 (2.93)	19.42 (5.03)	0.00 (2.85)	0.05 (2.88)
	Communication	32.26 (11.87)	1.32 (3.84)	0.63 (4.46)	32.32 (12.14)	−0.21 (5.35)	0.11 (4.42)	31.47 (11.70)	0.32 (3.76)	1.32 (4.88)
	Motivation	15.79 (6.27)	−0.21 (3.51)	0.00 (3.37)	15.16 (6.24)	0.42 (2.52)	0.63 (2.29)	15.26 (5.32)	0.05 (2.99)	1.11 (3.16)
	Autistic mannerisms	17.89 (6.03)	−0.37 (3.25)	−1.37 (3.27)	17.47 (7.19)	0.11 (4.24)	−0.68 (3.28)	17.26 (6.14)	−0.68 (3.58)	0.37 (2.50)

*Y-BOCS, Yale-Brown Obsessive Compulsive Scale; SRS, Social Responsiveness Scale*.

**Table 5:** **Adjusted estimate of behavioral outcome difference between baseline and post 8 h based on the mixed model**.

	**Post8h_baseline**									
**Outcomes**	**DLPFC vs. Sham**					**pSTS vs. Sham**				
	**Estimate (95% CI)**	**Degrees of freedom**	**t statistic**	***p*-value**	**Cohen's d**	**Estimate (95% CI)**	**Degrees of freedom**	**t statistic**	***p*-value**	**Cohen's d**
**PARTICIPANTS**										
Y-BOC: Obsession	−0.68 (−2.03 to 0.66)	34	−0.99	0.51	0.25	0.46 (−0.89 to 1.80)	34	0.67	0.73	0.27
Y-BOC: Compulsion	−0.58 (−2.02 to 0.85)	34	−0.8	0.64	0.26	−0.19 (−1.62 to 1.24)	34	−0.26	0.95	0.07
SRS total	−3.08 (−8.08 to 1.92)	34	−1.21	0.39	0.31	−5.24 (−10.24 to −0.24)	34	−2.05	0.09	0.71
Awareness	0.27 (−1.27 to 1.80)	34	0.34	0.92	0.14	0.19 (−1.34 to 1.73)	34	0.24	0.96	0.09
Cognition	0.38 (−1.23 to 1.99)	34	0.47	0.85	0.16	−0.28 (−1.88 to 1.33)	34	−0.34	0.92	0.12
Communication	−2.73 (−4.68 to −0.78)	34	−2.74	0.02^*^	0.76	−2.23 (−4.18 to −0.28)	34	−2.24	0.06	0.63
Motivation	−0.54 (−2.34 to 1.26)	34	−0.59	0.78	0.17	−1.38 (−3.18 to 0.42)	34	−1.51	0.24	0.48
Autistic mannerisms	−0.46 (−2.35 to 1.44)	34	−0.47	0.85	0.12	−1.54 (−3.44 to 0.35)	34	−1.59	0.21	0.53
**PARENTS**										
Y-BOCS: Compulsion	−0.49 (−1.96 to 0.98)	34	−0.65	0.74	0.38	−2.26 (−3.71 to −0.81)	34	−3.06	0.008^*^	0.89
SRS total	0.56 (−6.07 to 7.18)	34	0.16	0.98	0.05	−0.38 (−7.00 to 6.24)	34	−0.11	0.99	0.00
Awareness	0.30 (−1.05 to 1.65)	34	0.43	0.87	0.12	−0.79 (−2.14 to 0.56)	34	−1.15	0.42	0.38
Cognition	−0.78 (−2.45 to 0.90)	34	−0.91	0.57	0.28	0.07 (−1.60 to 1.75)	34	0.09	0.99	0.06
Communication	1.03 (−1.61 to 3.66)	34	0.76	0.67	0.26	−0.63 (−3.27 to 2.01)	34	−0.47	0.85	0.12
Motivation	−0.23 (−2.07 to 1.61)	34	−0.25	0.96	0.08	0.30 (−1.55 to 2.14)	34	0.32	0.93	0.13
Autistic mannerisms	0.24 (−1.66 to 2.13)	34	0.25	0.96	0.09	0.67 (−1.22 to 2.56)	34	0.69	0.71	0.20

The mixed model analysis was adjusted by sequence and period.

* *p < 0.05*.

**Table 6:** **Adjusted estimate of behavioral outcome difference between baseline and post 2 days based on the mixed model**.

	**Post2d_baseline**									
**Outcomes**	**DLPFC vs. Sham**					**pSTS vs. Sham**				
	**Estimate (95% CI)**	**Degrees of freedom**	**t statistic**	***p*-value**	**Cohen's d**	**Estimate (95% CI)**	**Degrees of freedom**	**t statistic**	***p*-value**	**Cohen's d**
**PARTICIPANTS**										
Y-BOC: Obsession	−0.14 (−1.36 to 1.07)	34	−0.23	0.96	0.02	0.69 (−0.52 to 1.91)	34	1.12	0.43	0.37
Y-BOC: Compulsion	−0.36 (−1.72 to 1.00)	34	−0.52	0.82	0.17	−0.17 (−1.53 to 1.19)	34	−0.25	0.96	0.07
SRS total	−4.69 (−10.65 to 1.28)	34	−1.54	0.23	0.44	−5.06 (−11.02 to 0.91)	34	−1.66	0.18	0.57
Awareness	−0.01 (−1.29 to 1.28)	34	−0.01	0.99	0.00	0.61 (−0.68 to 1.89)	34	0.92	0.56	0.25
Cognition	0.08 (−1.91 to 2.06)	34	0.07	0.99	0.05	−1.50 (−3.49 to 0.49)	34	−1.48	0.25	0.46
Communication	−2.72 (−5.23 to −0.21)	34	−2.13	0.07	0.63	−1.61 (−4.12 to 0.89)	34	−1.26	0.35	0.40
Motivation	−1.45 (−3.43 to 0.52)	34	−1.44	0.27	0.48	−1.18 (−3.16 to 0.80)	34	−1.17	0.41	0.46
Autistic mannerisms	−0.58 (−2.25 to 1.08)	34	−0.69	0.72	0.20	−1.37 (−3.03 to 0.30)	34	−1.61	0.20	0.49
**PARENTS**										
Y-BOCS: Compulsion	−1.25 (−3.04 to 0.53)	34	−1.38	0.30	0.52	−2.71 (−4.47 to −0.96)	34	−3.03	0.009^*^	0.85
SRS total	−4.49 (−10.95 to 1.98)	34	−1.36	0.31	0.38	−4.26 (−10.72 to 2.21)	34	−1.29	0.34	0.36
Awareness	0.01 (−1.34 to 1.36)	34	0.01	1.00	0.02	−1.53 (−2.88 to −0.19)	34	−2.23	0.06	0.77
Cognition	−0.84 (−2.65 to 0.97)	34	−0.91	0.57	0.28	0.39 (−1.42 to 2.20)	34	0.42	0.88	0.18
Communication	−0.76 (−3.51 to 1.98)	34	−0.55	0.81	0.15	−1.36 (−4.10 to 1.38)	34	−0.97	0.53	0.26
Motivation	−1.07 (−2.78 to 0.64)	34	−1.23	0.37	0.34	−0.55 (−2.26 to 1.16)	34	−0.63	0.75	0.17
Autistic mannerisms	−1.82 (−3.44 to −0.19)	34	−2.19	0.06	0.60	−1.20 (−2.83 to 0.43)	34	−1.45	0.27	0.36

The mixed model analysis was adjusted by sequence and period.

* *p < 0.05*.

For the repetitive behaviors measured by the Y-BOCS, scores of compulsive behaviors significantly decreased at 8 h (estimate = −2.26, *p* = 0.008, effect size = 0.89) and 2 days (estimate = −2.71, *p* = 0.009, effect size = 0.85) after the pSTS stimulation in comparison to the sham stimulation using the data from the reports of the parents. However, the results were not significant when the data from the reports of the participants were used. As for the comparison between the DLPFC and sham stimuli, there was no significant difference in either the self-reported or parent-reported Y-BOCS.

For the social outcomes measured by the SRS, self-reported scores of social communication significantly decreased at 8 h (estimate = −2.73, *p* = 0.02, effect size = 0.76), but not 2 days, after the iTBS over the DLPFC when compared to the sham treatment. However, no significant change was found when the data from the reports of parents were used. In addition, there was no significant improvement in both self-reported and parents' reported scores after iTBS over the pSTS when compared to the sham treatment.

### Adverse effects

Three participants felt transient discomfort during iTBS over the DLPFC because of muscle twitches around the eyes. No other adverse effect, such as a headache or seizure, was reported. Moreover, there was no obvious change in anxiety or mood symptoms observed using clinical assessment after iTBS.

## Discussion

Using a randomized, sham-controlled and crossover design, our pilot study investigated the impacts of iTBS on the DLPFC and pSTS in adults with ASD. We found that one session of iTBS over the bilateral DLPFC may alter the neuropsychological function in adults with ASD. The hit reaction time in the CCPT significantly decreased following the DLPFC session. Although we found commission errors in the CCPT and total errors in the WCST increased following the pSTS session, the results were not statistically significant.

### rTMS over the pSTS

The STS is important for social perception and social cognition (Zilbovicius et al., 2006). The anterior part of the STS is involved in voice processing (Kriegstein and Giraud, 2004), while the posterior part of the STS is important for analyzing biological motion (Pelphrey et al., 2003) and predicting the intention of others (Wyk et al., 2009). Previous studies have demonstrated the abnormal presentations of the pSTS in ASD (Boddaert et al., 2004; Redcay, 2008; Alaerts et al., 2014) and it has been proposed that the pSTS may be a therapeutic target in ASD (Saitovitch et al., 2016). However, the effect rTMS over pSTS on ASD has never been studied.

In the pilot study, we found a trend that iTBS over the pSTS may alter some neuropsychological functions in ASD, such as increasing commission errors in the CCPT and the total errors in the WCST. However, these findings were not statistically significant. Commission errors in the CCPT are known to relate to impulsivity, and the total errors in the WCST are related to cognitive flexibility. The findings may imply that, aside from the possible benefits on social behaviors, iTBS over the pSTS could impair some neuropsychological functions by increasing impulsivity and decreasing cognitive flexibility in ASD.

The results in behavioral outcomes were ambiguous. Although the parents reported significantly decreased compulsive behaviors in patients after iTBS (an excitatory protocol) over the pSTS, the patients did not report similar improvement. Besides, although the social communication and awareness improved after iTBS over the pSTS, the results were not statistically significant. Hence, the impacts of iTBS over the pSTS on behavior outcomes should be interpreted with caution, and a further study with a multi-sessional design may help for clarifying this point.

### rTMS over the DLPFC

Previous studies have demonstrated the possible therapeutic effects of rTMS over the DLPFC in ASD, including alterations in the event-related potential component in several brain areas (Sokhadze et al., 2009; Baruth et al., 2010), decreases in the omission errors in a modified three category oddball task (Sokhadze et al., 2012) and decreases in repetitive behaviors (Baruth et al., 2010; Sokhadze et al., 2010; Casanova et al., 2014). However, the social impairments observed in ASD did not improve (Baruth et al., 2010; Sokhadze et al., 2010).

Previous studies have found that rTMS over the DLPFC may alter several neuropsychological functions in healthy adults. Wagner found that one session rTMS over the left DLPFC (20 Hz) could significantly alter the visual divided attention while the results in Stroop test and WCST were unaffected in healthy adults (Wagner et al., 2006). In addition, Vanderhasselt demonstrated that one session high-frequency (10 Hz) rTMS over the DLPFC could decrease the reaction time in the Stroop test and set-switching test in healthy female adults (Vanderhasselt et al., 2006a,b). Moreover, the following studies demonstrated that rTMS over the DLPFC could alter the neuropsychological functions including the performance in digit span test (Aleman and van't Wout, 2008), the Tower of London task (van den Heuvel et al., 2013), and set-shift trials (Gerrits et al., 2015) in healthy adults.

In addition to healthy adults, rTMS over the DLPFC can also alter the neuropsychological functions in ASD. Sokhadze found that low-frequency rTMS over the DLPFC could significantly decrease the omission errors, commission errors and increase the reaction time in the Kanizsa illusory figure visual oddball task in ASD (Sokhadze et al., 2012, 2014). In contrast to previous studies in ASD, we found that single session iTBS over the DLPFC significantly reduced the reaction time in the CCPT but had no effect on omission and commission errors in the CCPT or any subscales in the WCST. The opposing results for reaction time might be explained by the stimulation protocol: the effect of low-frequency rTMS is inhibitory, while the iTBS used in our study is excitatory. In consistent with previous studies in healthy adults (Vanderhasselt et al., 2006a,b), we found that excitatory rTMS over the DLPFC could significantly decrease the reaction time of the neuropsychological tests in ASD.

### Mechanism of action of rTMS in ASD

Most studies have demonstrated that low-frequency rTMS improves some deficits in ASD. Low-frequency rTMS is thought to induce long-term depression (Hoffman and Cavus, 2002). Event-related potential studies have indeed confirmed that the benefit of low-frequency rTMS over the DLPFC comes from increasing the activation of inhibitory circuits in ASD (Sokhadze et al., 2009, 2010; Baruth et al., 2010; Casanova et al., 2012).

Interestingly, we found that excitatory TBS over the DLPFC also altered the neuropsychological function in ASD. Previous studies have shown that the balance of cortical excitation and inhibition is abnormal in ASD (Casanova et al., 2002; Yizhar et al., 2011). This imbalance contributes to abnormalities in the cortical minicolumns, especially within the prefrontal cortex (Casanova, 2006). iTBS is known to enhance both the excitatory and inhibitory circuits beneath the coil (Huang et al., 2005, 2011). Therefore, we propose that iTBS enhances some inhibitory circuits that are required to improve social function in ASD. Moreover, the study of descending volleys of rTMS showed that iTBS enhanced mainly the later I waves, while 1 Hz rTMS suppressed mainly the I1 wave, indicating that iTBS and low-frequency rTMS may activate different circuits (Di Lazzaro et al., 2010). This may explain why the effect of iTBS is not simply opposite to the effect of low-frequency rTMS over the DLPFC.

This pilot study has several limitations. First, the sample size is relatively small, although it is comparable to that used in previous studies. Thus, the effect of iTBS over the pSTS or DLPFC may not be obvious. However, even with the small subject numbers, we still found the significant effect of iTBS over the pSTS or DLPFC on behavioral and neuropsychological outcomes compared to the sham treatment. The second limitation is the measurement of neuropsychological function. In the pilot study, we used only the CCPT and WCST, which are not directly related to the function of pSTS, to evaluate executive abilities because limited tools were available when the study was initiated. Other fields of neuropsychological function such as social cognition or biological motion detection should be considered as measures for the effect of pSTS stimulation in future studies. The third limitation is the measurement tool used to evaluate the behavioral symptoms. Consistent with previous studies, we used the SRS to measure the impact of rTMS (Sokhadze et al., 2009, 2010; Baruth et al., 2010; Casanova et al., 2012). However, the SRS was designed to evaluate the social ability of participants in the past 6 months, and may not be sensitive enough to capture changes over very short time periods. The development of better measurements to assess the impact of rTMS in ASD is important and will be necessary for the future (Oberman et al., 2016). The fourth limitation is the time window to evaluate the changes in behaviors and social symptoms in ASD. One early study demonstrated that the aftereffect of rTMS on compulsive behaviors could be found 8 h afterward (Greenberg et al., 1997). Following studies demonstrated that the effect of one session rTMS does not last for 1 week, and a week interval has been commonly applied to control the residual effect in a within-subject design (Simis et al., 2013; Kim and Shin, 2014; Tard et al., 2016). Based on these findings, we decided to evaluate the changes in behaviors and social symptoms in ASD 8 h and 2 days afterward. However, it is indeed arguable that the effect on behaviors and social symptoms may last longer than the period that we followed up. Longer time window to evaluate the changes in behavior and social symptoms should be considered in the future studies. To minimize the possible residual effect that may affect the effect of the subsequent stimulation, the order of the interventions was controlled and counterbalanced, and a mixed model analysis was used. The final limitation is the possibility of observational bias and placebo effects. Several steps were adopted to decrease these risks: the performance of iTBS and the data analysis were completed by different people; parents were not present during iTBS so that they would not know the stimulation location during each session; a crossover design and sham control treatment was applied to reduce the possibility of placebo effects.

This pilot study has several strengths. First, by adopting a crossover design, we were able to clearly compare the effect of iTBS over the DLPFC, the pSTS and the sham control in the same individuals. Second, the behavioral outcomes were measured using different resources, including both the participants themselves and their parents. Third, the localization of the DLPFC and pSTS via the process of coordinate transformation and the TMS-dedicated navigation system used is more precise than those in previous studies where no navigator was used.

In conclusion, this study revealed that the neuropsychological function in ASD was modified by the iTBS intervention. However, the effect of iTBS over either the DLPFC or the pSTS on behaviors in adults with ASD remains inconclusive and deserves further evaluation. To our knowledge, this is the first study to demonstrate the impacts of a facilitatory intervention over the pSTS in adults with ASD. Our findings indicate that the pSTS could be a new intervention target for ASD; thus, further long-term investigations should be performed in the future.

## Author contributions

HN, JH, CW, YW, RC, and YH participated in the study concept and design. HN and YW were involved in subjects recruitment and assessment. CW participated in acquisition of imaging data. HN did the iTBS intervention. HN and CC were involved in data analysis and interpretations. HN and JH were responsible for Table and figure production. HN, JH, and YH were involved in the writing of manuscript. All authors read and approved this manuscript.

### Conflict of interest statement

The authors declare that the research was conducted in the absence of any commercial or financial relationships that could be construed as a potential conflict of interest. The reviewer AS and handling Editor declared their shared affiliation, and the handling Editor states that the process nevertheless met the standards of a fair and objective review.
